# Supplementary material for: Evaluating the documentation of vital signs following implementation of a new comprehensive newborn monitoring chart in 19 hospitals in Kenya: A time series analysis
Source: PLOS Glob Public Health. 2023 Nov 1;3(11):e0002440. doi: 10.1371/journal.pgph.0002440 (PMC10619831; doi:10.1371/journal.pgph.0002440)
Supplement: S1 Appendix — (DOCX) [file pgph.0002440.s002.docx]

# Hospital characteristics

| **Hospital Code** | **CIN-N Join Date** | **Total Patients** | **Study Months** | **Median Monthly Patient Count^a^** | **CIN audit site** | **NEST site** |
| --- | --- | --- | --- | --- | --- | --- |
| H1 | Nov-18 | 4551 | 24 | 92 (83-98) | No | Yes |
| H2 | Feb-18 | 3165 | 24 | 48 (43-54) | No | No |
| H3 | Mar-18 | 5755 | 24 | 100 (93-114) | No | Yes |
| H4 | Jun-19 | 865 | 23 | 20 (17-22) | No | No |
| H5 | Nov-18 | 3891 | 24 | 73 (60-82) | No | Yes |
| H6 | Apr-18 | 6419 | 24 | 102 (96-110) | No | Yes |
| H7 | Mar-18 | 5029 | 24 | 82 (76-91) | No | Yes |
| H8 | Oct-18 | 9169 | 24 | 186 (177-213) | Yes | Yes |
| H9 | Mar-18 | 2270 | 23 | 39 (27-43) | No | No |
| H10 | Mar-18 | 10092 | 24 | 148 (133-154) | No | Yes |
| H11 | Jun-17 | 10062 | 24 | 147 (136-172) | Yes | Yes |
| H12 | Mar-18 | 4524 | 24 | 61 (44-76) | No | No |
| H13 | Nov-19 | 2840 | 22 | 77 (67-81) | No | No |
| H14 | Sep-19 | 6674 | 24 | 145 (132-154) | No | Yes |
| H15 | Apr-18 | 4742 | 24 | 67 (59-74) | No | Yes |
| H16 | Apr-14 | 33561 | 24 | 280 (217-313) | Yes | Yes |
| H17 | Oct-18 | 8567 | 24 | 157 (138-174) | No | Yes |
| H18 | Mar-18 | 1843 | 24 | 28 (21-36) | No | No |
| H19 | Jul-17 | 1314 | 24 | 26 (21-31) | No | No |
| ^a^ Monthly patient counts is for all patients admitted to the newborn ward since joining CIN-N | | | | | | |
