# Supplementary material for: Evaluating the documentation of vital signs following implementation of a new comprehensive newborn monitoring chart in 19 hospitals in Kenya: A time series analysis
Source: PLOS Glob Public Health. 2023 Nov 1;3(11):e0002440. doi: 10.1371/journal.pgph.0002440 (PMC10619831; doi:10.1371/journal.pgph.0002440)
Supplement: S2 Appendix — (DOCX) [file pgph.0002440.s003.docx]

# Appendix 2 Detailed tables and charts.

Table 1 Comparison table TPRS vs TPRS categorical outcome pre- and post-intervention average (19 hospitals)

|  | **T-P-R-S Monitoring** | | **T-P-R Monitoring** | |
| --- | --- | --- | --- | --- |
| **Category** | Before (N=22023)  n (%) | After (N=21696)  n (%) | Before(N=22023)  n (%) | After (N=21696)  n (%) |
| No Monitoring 0 | 15089 (68.5%) | 9434 (43.5%) | 12058 (54.8%) | 7180 (33.1%) |
| Monitoring 1 to 3 times | 2503 (11.4%) | 4915 (22.7%) | 4002 (18.2%) | 5583 (25.7%) |
| Monitoring 4 to 7 times | 1669 (7.6%) | 3573 (16.5%) | 2578 (11.7%) | 4576 (21.1%) |
| Monitoring 8 times or more | 2762 (12.5%) | 3774 (17.4%) | 3385 (15.4%) | 4357 (20.1%) |

Fig 1 TPRS vs TPRS categorical outcome at the hospital level


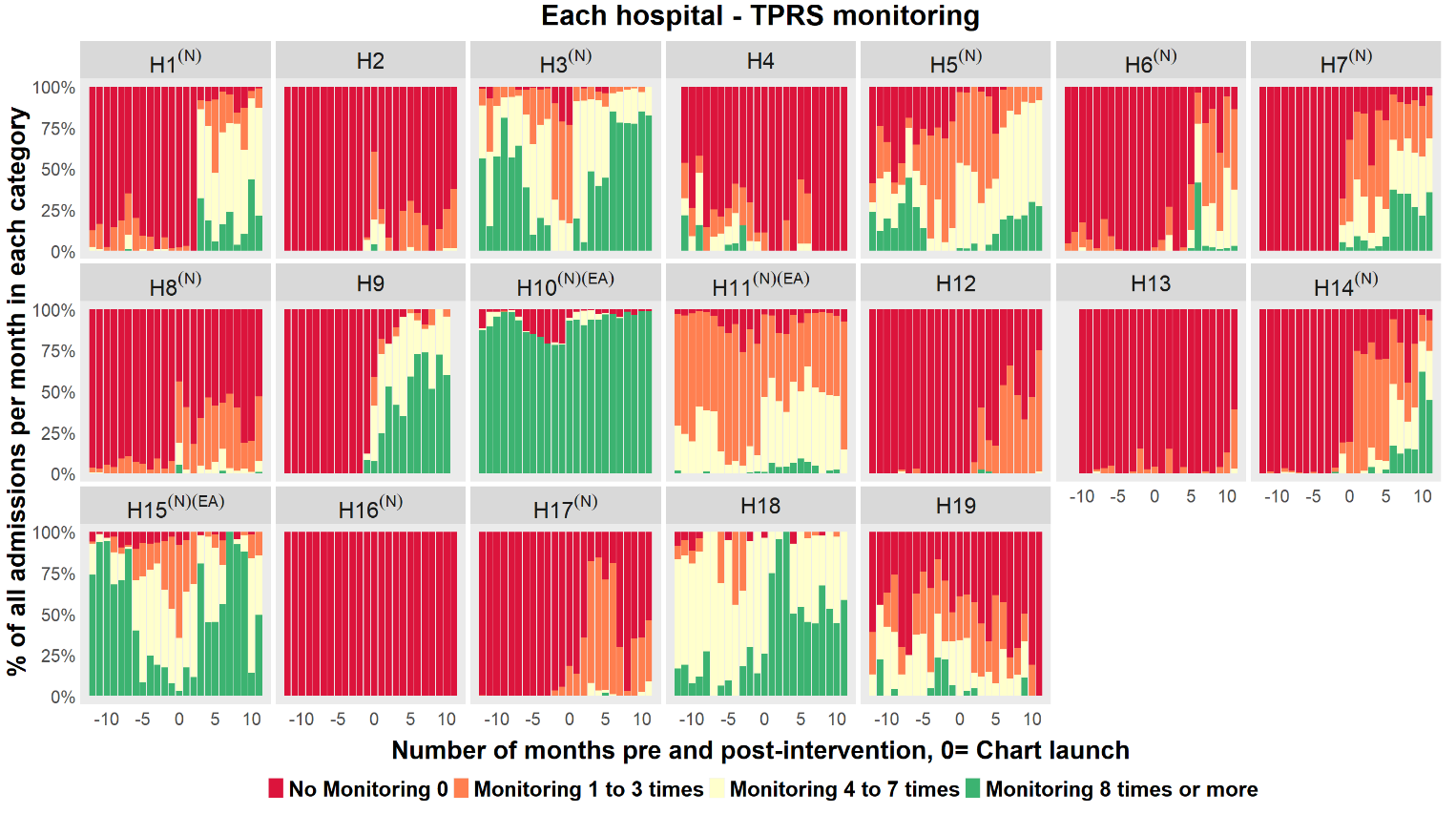


Proportions of patients monitored in each category over time per hospital TPRS composite outcome.


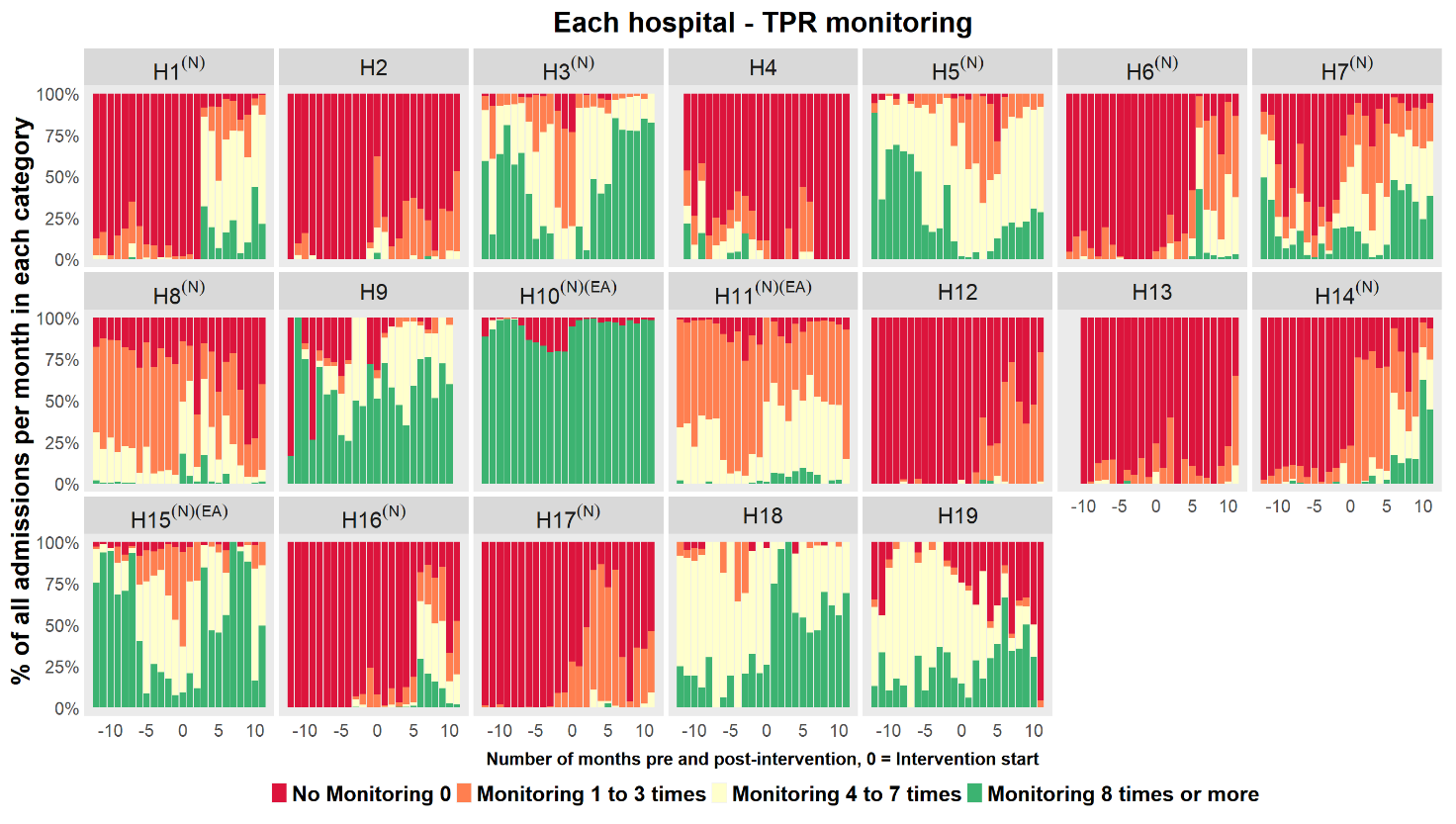


Proportions of patients monitored in each category over time per hospital TPR composite outcome.


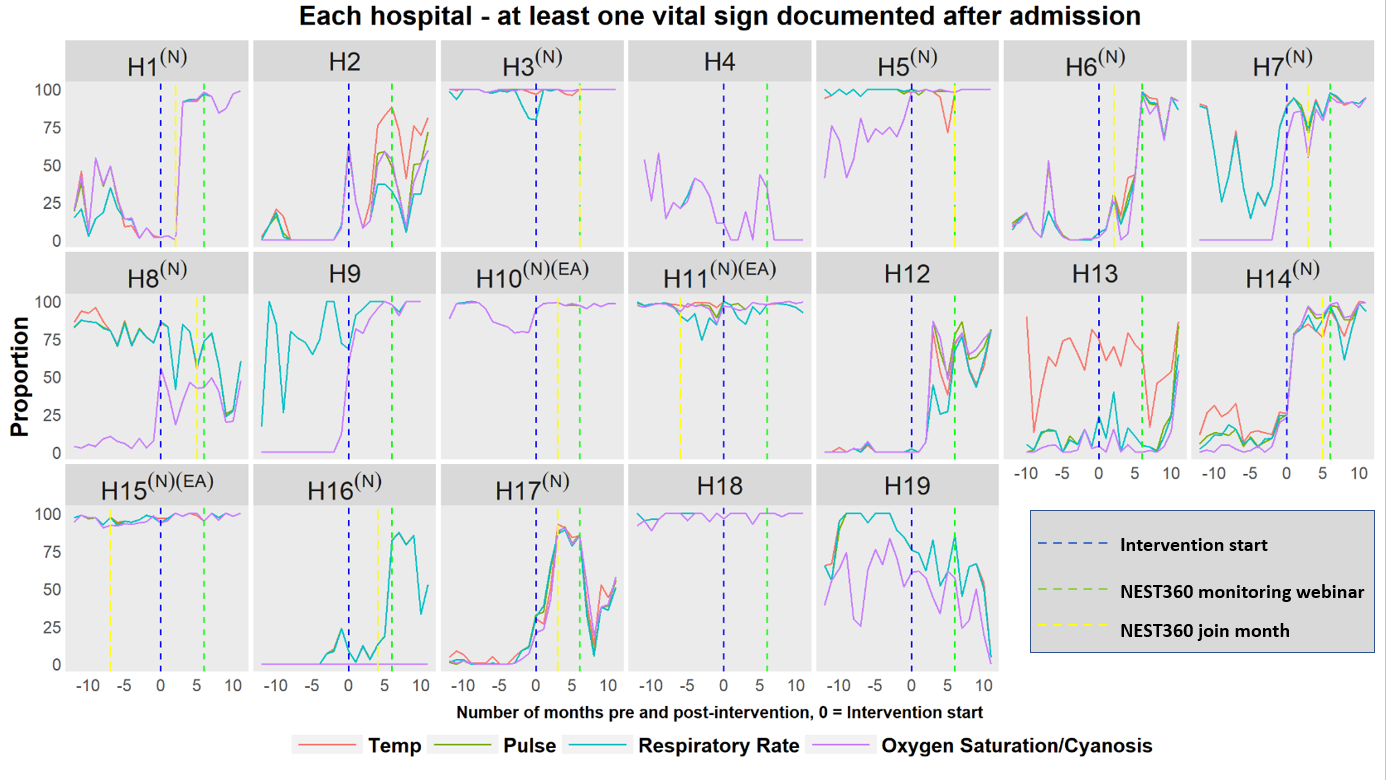


Fig 2 At least one vital sign documented over time per hospital.
