# Supplementary figures and images for: Evaluating the documentation of vital signs following implementation of a new comprehensive newborn monitoring chart in 19 hospitals in Kenya: A time series analysis
Source: PLOS Glob Public Health. 2023 Nov 1;3(11):e0002440. doi: 10.1371/journal.pgph.0002440 (PMC10619831; doi:10.1371/journal.pgph.0002440)

# Appendix 3 Chart review sample images


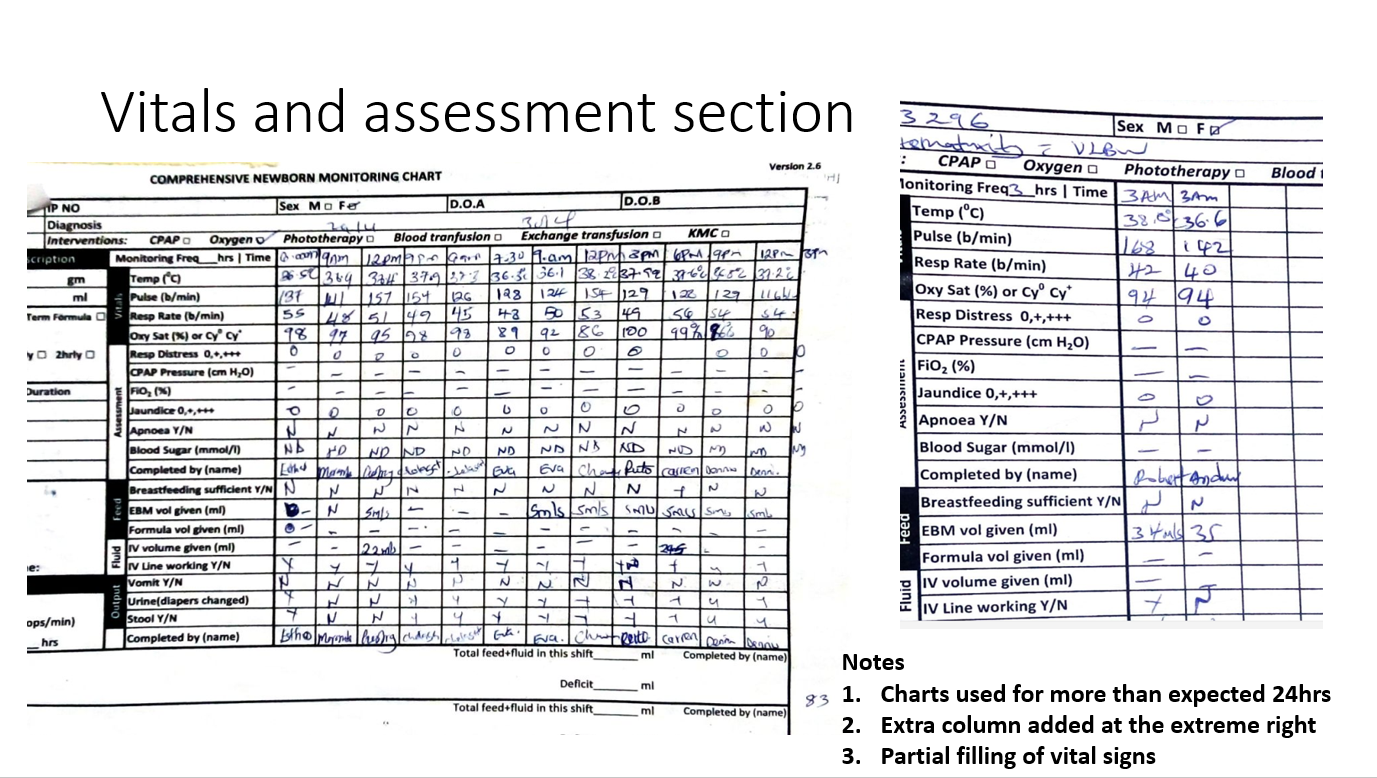

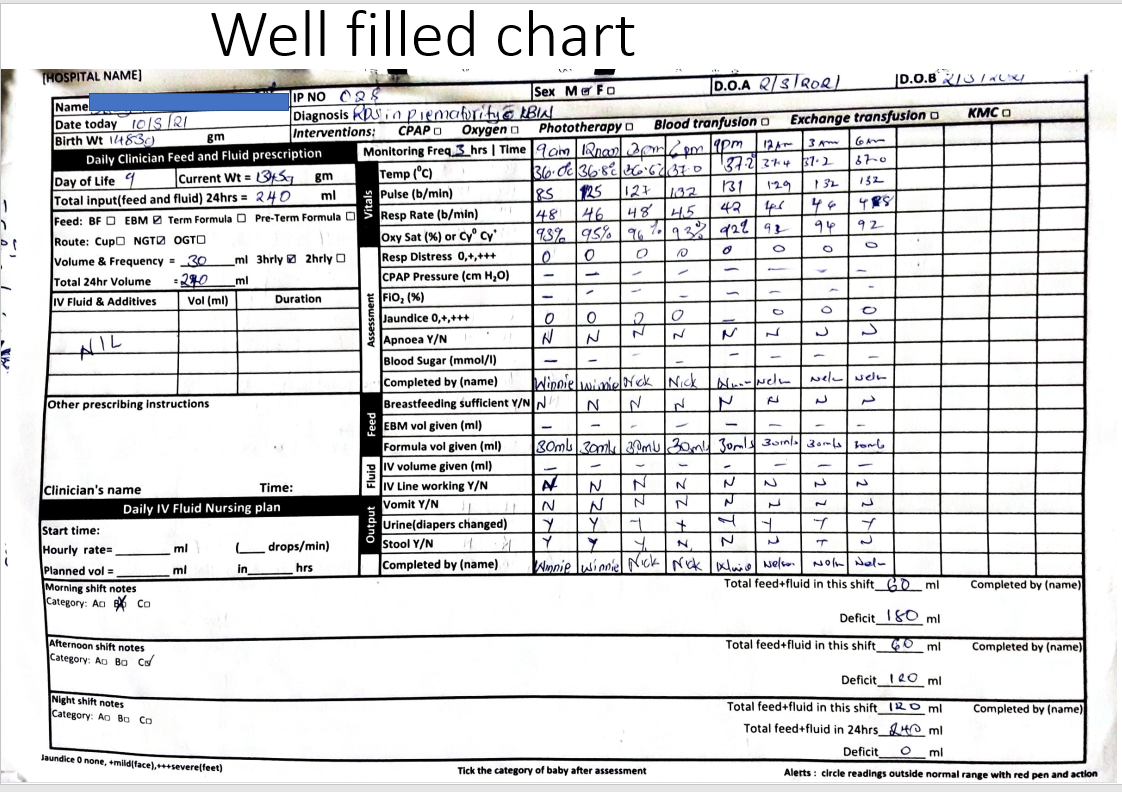

Supplement: S3 Appendix — (DOCX) [file pgph.0002440.s004.docx]
