# Supplementary material for: Evaluating the documentation of vital signs following implementation of a new comprehensive newborn monitoring chart in 19 hospitals in Kenya: A time series analysis
Source: PLOS Glob Public Health. 2023 Nov 1;3(11):e0002440. doi: 10.1371/journal.pgph.0002440 (PMC10619831; doi:10.1371/journal.pgph.0002440)
Supplement: S4 Appendix — (DOCX) [file pgph.0002440.s005.docx]

# S4 Appendix 4 Interaction model results

Table 1: Adjusted odds ratios and p-values of models with interaction between step change (Intervention) and slope change (Trend) with severity of illness (SENSS score)

|  | **Respiratory rate** | | **Oxygen Saturation / Cyanosis** | | **Temperature** | | **TPRS** | |
| --- | --- | --- | --- | --- | --- | --- | --- | --- |
|  | aOR (CI) | P-value | aOR (CI) | P-value | aOR (CI) | P-value | aOR (CI) | P-value |
| **Intervention** | 1.79 (1.62-1.98) | <0.001 | 5.64 (4.97-6.39) | <0.001 | 1.60 (1.45-1.76) | <0.001 | 4.93 (4.33-5.60) | <0.001 |
| **Pre-intervention trend** | 0.96 (0.95-0.97) | <0.001 | 0.94 (0.93-0.95) | <0.001 | 0.93 (0.92-0.94) | <0.001 | 0.96 (0.95-0.97) | <0.001 |
| **Change in trend** | 1.17 (1.15-1.19) | <0.001 | 1.21 (1.19-1.24) | <0.001 | 1.20 (1.18-1.21) | <0.001 | 1.16 (1.14-1.18) | <0.001 |
| **SENSS score** | 1.07 (1.03-1.11) | <0.001 | 1.08 (1.03-1.13) | 0.002 | 1.06 (1.02-1.09) | 0.002 | 1.13 (1.07-1.18) | <0.001 |
| **Intervention x SENSS score** | 0.91 (0.84-0.99) | 0.022 | 0.91 (0.83-1.00) | 0.045 | 0.94 (0.87-1.02) | 0.132 | 0.83 (0.75-0.91) | <0.001 |
| **Trend x SENSS score** | 1.04 (1.03-1.05) | <0.001 | 1.03 (1.02-1.04) | <0.001 | 1.04 (1.03-1.05) | <0.001 | 1.04 (1.02-1.05) | <0.001 |
| **In NEST Program** | 2.22 (2.02-2.43) | <0.001 | 1.87 (1.68-2.08) | <0.001 | 2.29 (2.10-2.50) | <0.001 | 2.24 (2.01-2.50) | <0.001 |

The x-axis spans the inter-quantile range of scaled SENSS score (-0.78 to 0.5). The odds ratios of step change (Intervention) decrease with severity of illness, while the odds ratios or change in slope (Trend) increase with severity of illness.


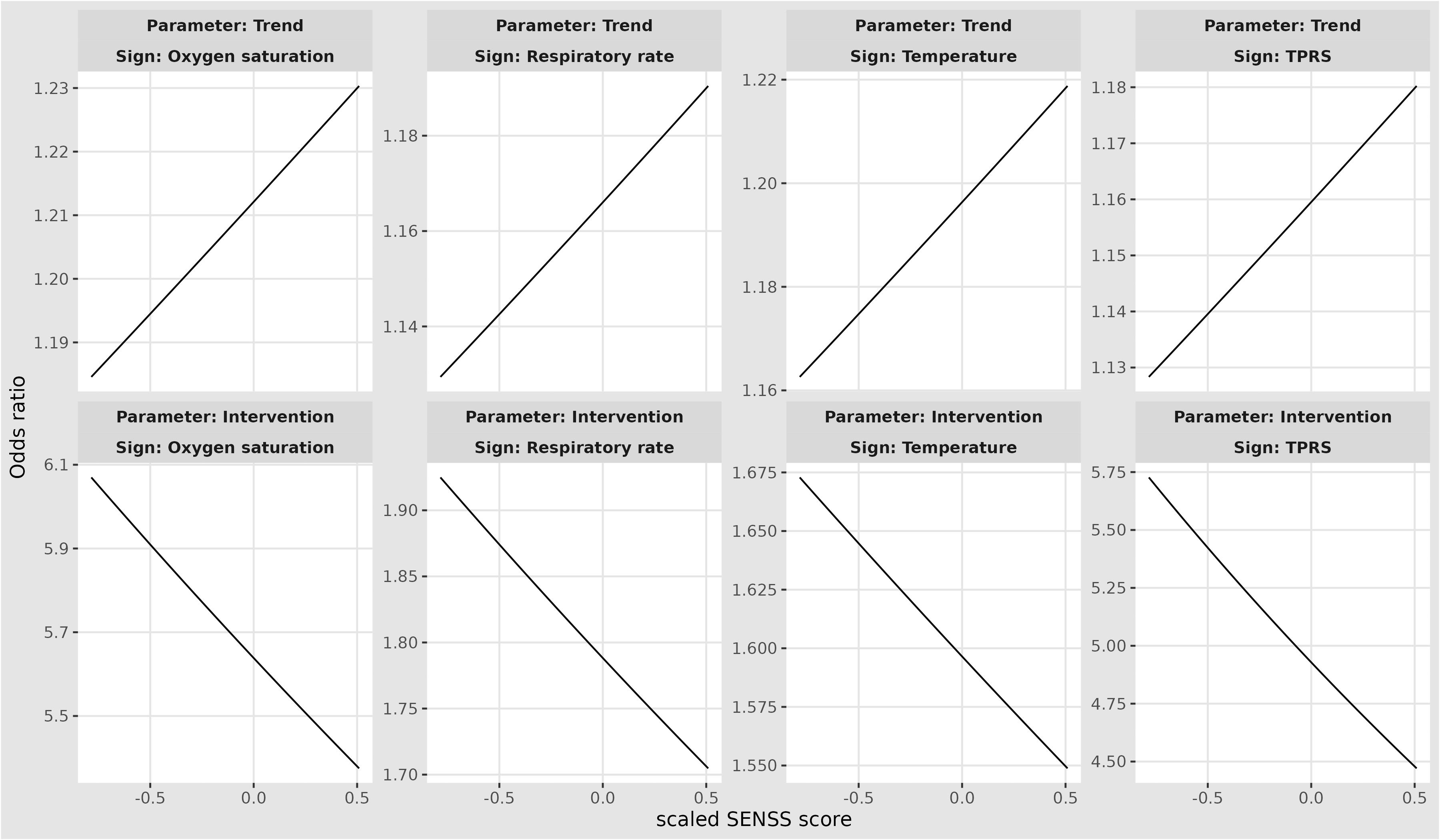


Fig 1 Odds ratio of step change (Intervention) and slope change (Trend) against severity of illness (scaled SENSS score).
