# Supplementary material for: Evaluating the documentation of vital signs following implementation of a new comprehensive newborn monitoring chart in 19 hospitals in Kenya: A time series analysis
Source: PLOS Glob Public Health. 2023 Nov 1;3(11):e0002440. doi: 10.1371/journal.pgph.0002440 (PMC10619831; doi:10.1371/journal.pgph.0002440)
Supplement: Attachment — Submitted filename: Response to reviewers.docx [file pgph.0002440.s007.docx]

Dear editor/reviewers,

Thank for taking time to read our manuscript and provide constructive comments that have helped make it better. We provide inline a point by point response to the comments.

| Journal Requirements:  1. Please include a complete copy of PLOS’ questionnaire on inclusivity in global research in your revised manuscript. Our policy for research in this area aims to improve transparency in the reporting of research performed outside of researchers’ own country or community. The policy applies to researchers who have travelled to a different country to conduct research, research with Indigenous populations or their lands, and research on cultural artefacts. The questionnaire can also be requested at the journal’s discretion for any other submissions, even if these conditions are not met.  Please find more information on the policy and a link to download a blank copy of the questionnaire here: <https://journals.plos.org/globalpublichealth/s/best-practices-in-research-reporting>. Please upload a completed version of your questionnaire as Supporting Information when you resubmit your manuscript. | A completed copy of PLOS’ questionnaire on inclusivity in global research is included in the supporting information files(S5) |
| --- | --- |
| **Reviewer #1** | |
| Thank you for the opportunity to review this interesting paper! I really like this paper — it addresses an important gap in health care quality, gives a lot of rich contextual information about the study setting, describes an interesting intervention, and includes very rich data on the effects of the intervention. I have several questions and suggestions for strengthening the paper, mainly focused on the interrupted time-series analysis. Please see below: | Thank you for the compliments and for taking time to review the manuscript in detail. |
| 1. Did the intervention include any quality-checks on the data reported on the charts? Is there a way to know whether the charts were actually used as intended, vs. completed at the end of the day by a nurse? (If no - perhaps future work could include some direct observation of care to document whether charts are used as intended). | The intervention did not include a check on actual data entered on the charts. However, a qualitative audit of a sample of 86 filled charts showed that the charts were used variably as described in the qualitative audit of charts section.  We agree that future work should include an assessment of actual values and have added a subtitle to the last paragraph of the discussion section to highlight this. |
| 2. Do you have any information about whether the intervention led to increased monitoring of vital signs, as opposed to only increased documentation of monitoring? In some places in the manuscript you talk about ‘higher monitoring’ but in others you talk mainly about better documentation. It would be interesting to understand more about whether the charts actually encourage providers to check vital signs more frequently vs. giving them a clearer/more organized way to record vital signs information. | Thank you for this comment. We have clarified the text throughout to read : the odds of the vital signs being documented in a higher monitoring category.  Regarding the vital signs being checked more frequently, it is possible that this was happening but not documented. In the qualitative audit of charts this is mentioned briefly as well as under limitations, where we have indicated that there were other monitoring charts available as well as other places that the staff could have documented the vital signs.  We agree that a better way to capture this would be through a direct observational study as suggested in point 1 above. |
| 3. To better understand the analysis and the interpretation of the results, it would be helpful if you could include a regression equation in the manuscript. | Thank you for this comment. The equation is now included in the methods section. |
| 4. The authors chose to use a logistic regression, but this model could have also potentially been fit as a linear probability model, which would have been easier to interpret. The logistic regression assumes that the trend over time in vital signs documentation is linear in the log odds…did you find this to be the case? It would be helpful to include a justification for the use of logistic regression here, and to include results for a linear probability model as a sensitivity analysis. | For each of 4 vital signs (TPRS) the number of times monitored was recorded to 10 then truncated anything above 10, so it is possible a very sick baby with very many vital signs measured would not have them all counted. This plus the fact that there is high correlation between TPRS counts – as expected from knowledge of nursing practice – raised concerns to us about treating the outcome as a linear outcome measure. We therefore, felt it was more sensible therefore to use a categorical outcome in our model as described in the methods section. |
| 5. I found some of the phrasing of the results to be fairly confusing. For example, “In the pre-intervention period, the odds of being in a higher monitoring category were declining by 4% every month while the change in the odds of being in a higher monitoring category increased by 16 per month in the post-intervention period.” If I understand the model correctly, I think you could instead write this as, “In the pre-intervention period, the odds of being in a higher monitoring category were declining by 4% every month. Following the intervention, there was a change in this trend: the odds began to increase by 12% per month.” (This is assuming that the model is something like y = a + b1*month + b2*post + b3*month*post, and b1=-0.04, and b2=0.16.) There are several places where I feel that the language describing the results could be clarified in this way. | Thank you for this suggestion. We have modified the text in the abstract and results section to make it clearer. |
| 6. Figure 2- why does it look like vital signs documentation decreased after the webinar? | Thank you for this comment. This refers to figure 3. The vertical line should be at month 5 post intervention and not month 6 as was shown. We have corrected this in the charts  We have also clarified in Line 94 that the intervention start month is September 2020 owing to different start dates(explained from line 393)  The chart launch month was July 2020  We have also updated the correct bar charts with the legend showing: month 0 = Intervention start (figure 2) |
| 7. In the analysis, you adjust for how sick the baby is. By including this variable in the model, you essentially allow for a higher intercept (more vital signs documentation) for the sicker babies. However, I think it would be interesting to know whether the intervention actually worked better (or worse) for these babies. To do this, you could include an interaction between the “post” variable for the intervention and the sickness variable. | We have added a model that includes interaction terms to assess effect modification by severity of illness. The model specification has been added to the methods section and the outcome in the results section. We have also included an appendix with the relevant table and charts. |
| 8. In the introduction, it would be useful to have an additional 1-2 sentences describing why documentation of vital signs is so important in neonatal wards. (You reference previous work, which explains this well, but I think that some readers may miss this and it could be stated more clearly upfront in this manuscript). | Thank you for this comment. We have added a statement in the introduction to make this more explicit. |
| **Reviewer #2** | |
| I appreciate the opportunity to review this manuscript. The authors' effort to improve the quality of care for sick newborns through more frequent monitoring of vital signs is highly commendable. The study was done according to high methodological standards. I see no significant methodological or formal errors in the manuscript. It is well-written and has lots of essential information. It was easy to follow with a good explanation of the study rationale, a reference to the design process of the monitoring chart used, a clear presentation of results, and a discussion of findings leading to a sound conclusion. Overall, the study has value, and I will recommend it for publication. I have some minor queries you might find helpful in progressing your study report toward publication. | Thank you for taking time to review the manuscript and providing suggestions to improve it. We appreciate the compliments. |
| The description of the study design could have been more explicit. Were there any qualitative scoping activities (i.e. interviews or FGDs)? These were not mentioned in the methodology or the result sections. | Thank you for this comment. The study is an Interrupted time series analysis of the quantitative data that is complemented by a qualitative audit of filled charts. This is described in the Methods as well as results sections. There were no KIIs or FGDs in this study. |
| I note some quotes from nurses in the last paragraph of the discussion section. Are these from KIIs/FGDs? Please clarify. | Thank you for this comment. The last paragraph of the discussion references our previous interactions with nurses which are also captured in detail in the referenced manuscript (33). We have added the word ‘prior’ to clarify this and not confuse the readers. |
| Considering the above, it may be worth revisiting your description of the study design- description of methods. Should it be quantitative rather than a mixed design? The same reason why I found the use of the term "Qualitative audit of monitoring charts" to describe a review of the quality of documentation of vital signs a misnomer. Please clarify | Thank you for this comment. we have revised the methods section to clarify that this was a quantitative study. |

Sincerely,

Naomi Muinga

Corresponding author
